# Supplementary material for: Co-occurring expression and methylation QTLs allow detection of common causal variants and shared biological mechanisms
Source: Nat Commun. 2018 Feb 23;9:804. doi: 10.1038/s41467-018-03209-9 (PMC5824840; doi:10.1038/s41467-018-03209-9)
Supplement: Supplementary file 1 — Supplementary Information [file 41467_2018_3209_MOESM1_ESM.pdf]

Supplementary Information

Co-occurring expression and methylation QTLs allow detection of common causal variants and shared biological mechanisms

Pierce, et al.

Supplementary Table 1. Logistic regression of Sobel P (SME) <0.05, Sobel P (SEM) <0.05, and Partial correlation P<0.05 on eQTL/meQTL P-values

Predictors	Outcome					
	Sobel P (SME) <0.05		Sobel P (SEM) <0.05		Corr. P<0.05	
	OR (95%CI)	p-value	OR (95%CI)	p-value	OR (95%CI)	p-value
**cis-eQTL P-value quartiles**						
Quartile 1 (largest P-value)	1	Ref	1	Ref	1	Ref
Quartile 2	1.63 (1.10, 2.42)	0.02	3.38 (1.84, 6.22)	<.0001	1.32 (0.95, 1.82)	0.1
Quartile 3	2.61 (1.80, 3.77)	<.0001	7.56 (4.27, 13.40)	<.0001	1.86 (1.36, 2.53)	<.0001
Quartile 4 (smallest P-value)	2.91 (2.02, 4.19)	<.0001	9.86 (5.61, 17.33)	<.0001	2.21 (1.64, 3.00)	<.0001
**cis-meQTL P-value quartiles**						
Quartile 1 (largest P-value)	1	Ref	1	Ref	1	Ref
Quartile 2	1.41 (0.95, 2.08)	0.09	1.38 (0.90, 2.11)	0.14	1.15 (0.84, 1.58)	0.39
Quartile 3	2.02 (1.40, 2.92)	0.0002	1.99 (1.33, 2.96)	0.0008	1.45 (1.07, 1.97)	0.02
Quartile 4 (smallest P-value)	3.04 (2.14, 4.31)	<.0001	2.65 (1.80, 3.90)	<.0001	2.06 (1.54, 2.77)	<.0001

The sample size consists of 2,913 gene expression probe-SNP-CpG trios with posterior probability of one common causal variant >0.80.

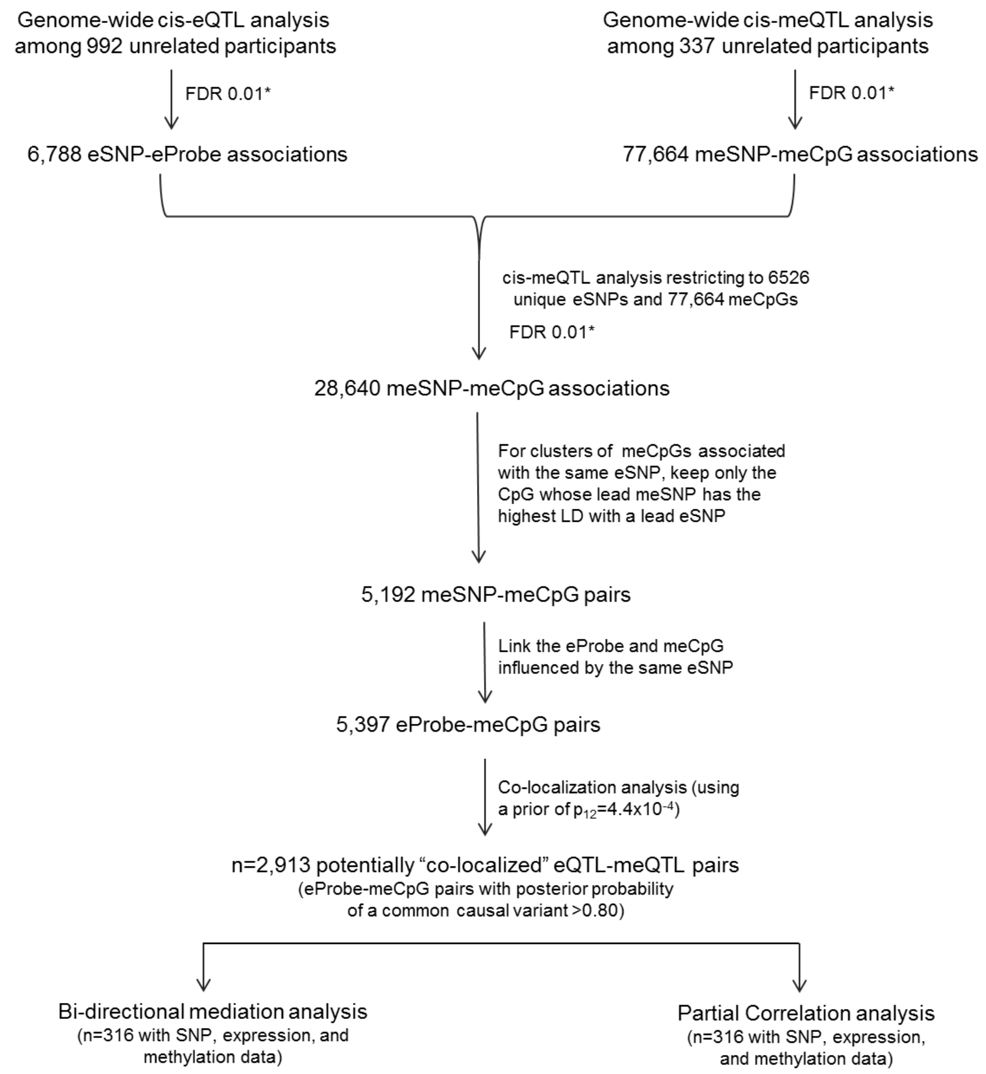

Supplementary Figure 1. Workflow for identifying eQTLs, meQTLs, and co-localized eQTL-meQTL pairs. *Beta distribution-adjusted empirical p-values from FastQTL were used to calculate q-values (Storey & Tibshirani, PNAS, 2003), and a false discovery rate (FDR) threshold of <0.01 was applied at the probe level to identify eProbes/meCpGs with a significant eQTL/meQTL.

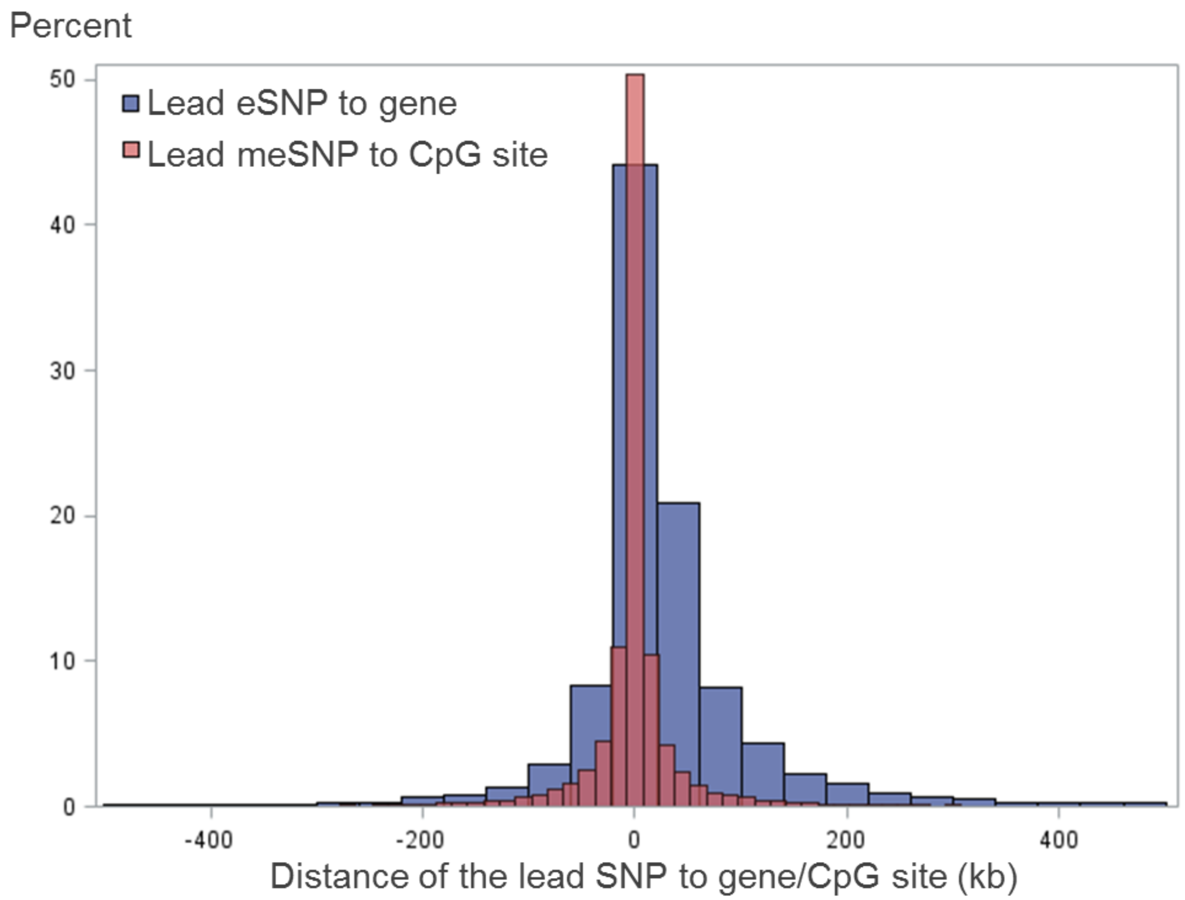

Supplementary Figure 2. Distance of the lead eSNP to the transcript start site of the target gene (n=6,788) and the distance of the lead meSNP to the affected CpG site (n=77,664).

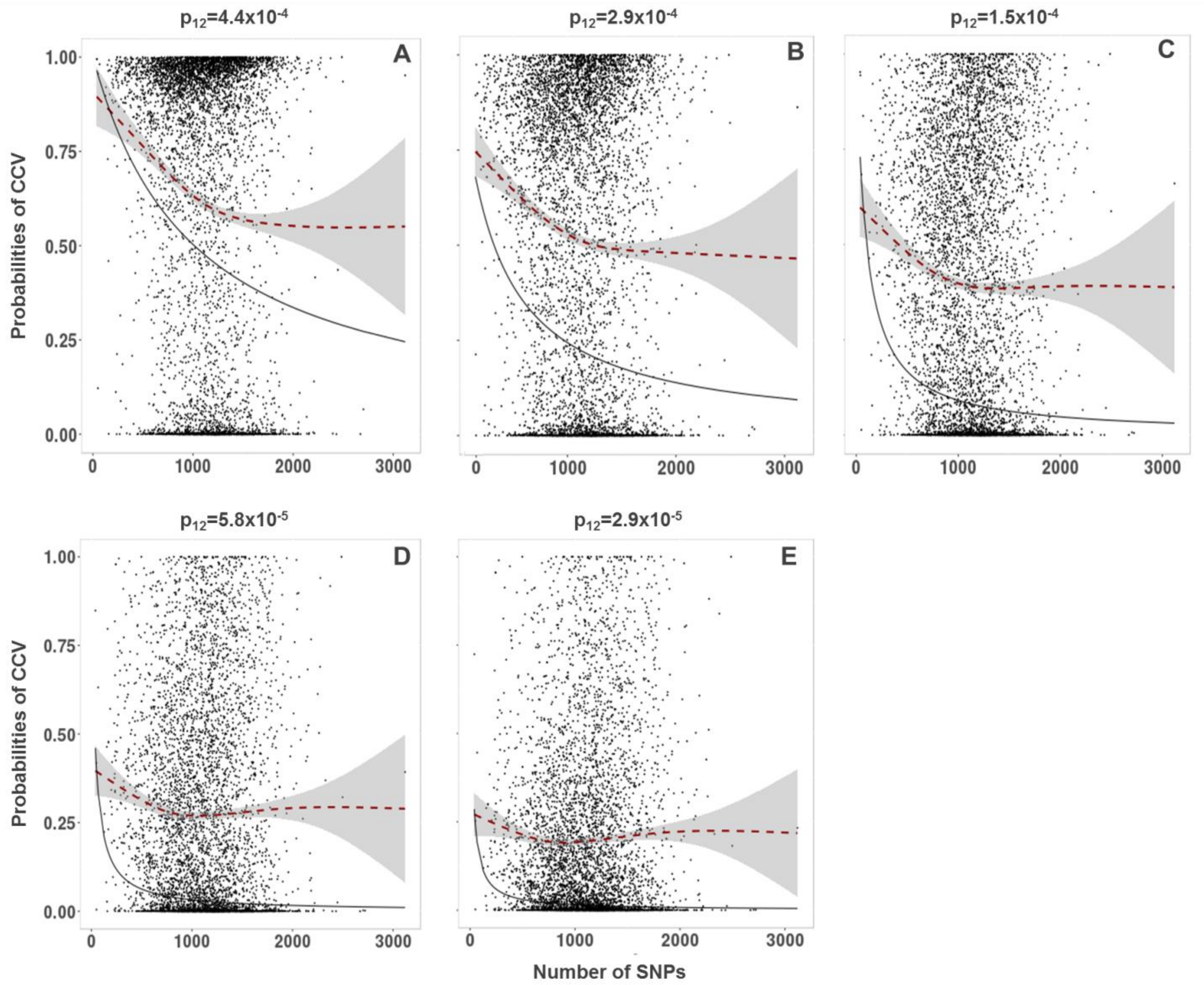

Supplementary Figure 3. Effect of choice of prior probability (p_{12}) of common causal variant (CCV) on the posterior probability of a CCV. For each eQTL-meQTL pair undergoing co-localization analyses, we plotted the relative posterior support for a CCV (as opposed to distinct causal variants, DCV) for each co-localization test, defined by PP of CCV/(PP of DCV + PP of CCV), against the number of SNPs used for each co-localization test . The red dotted line is the Loess smoothing curve of these points. The black solid line is the relative prior support for a CCV, based on the values selected the priors (p_1, p_2, and p_{12}), calculated using the equation provided by Guo *et al.*[22] This data is restricted to co-localization tests with a posterior probability of two distinct causal variants (PP of DCV) + posterior probability of common causal variant (PP of CCV) > 0.8 (100 out of 5397 tests excluded).

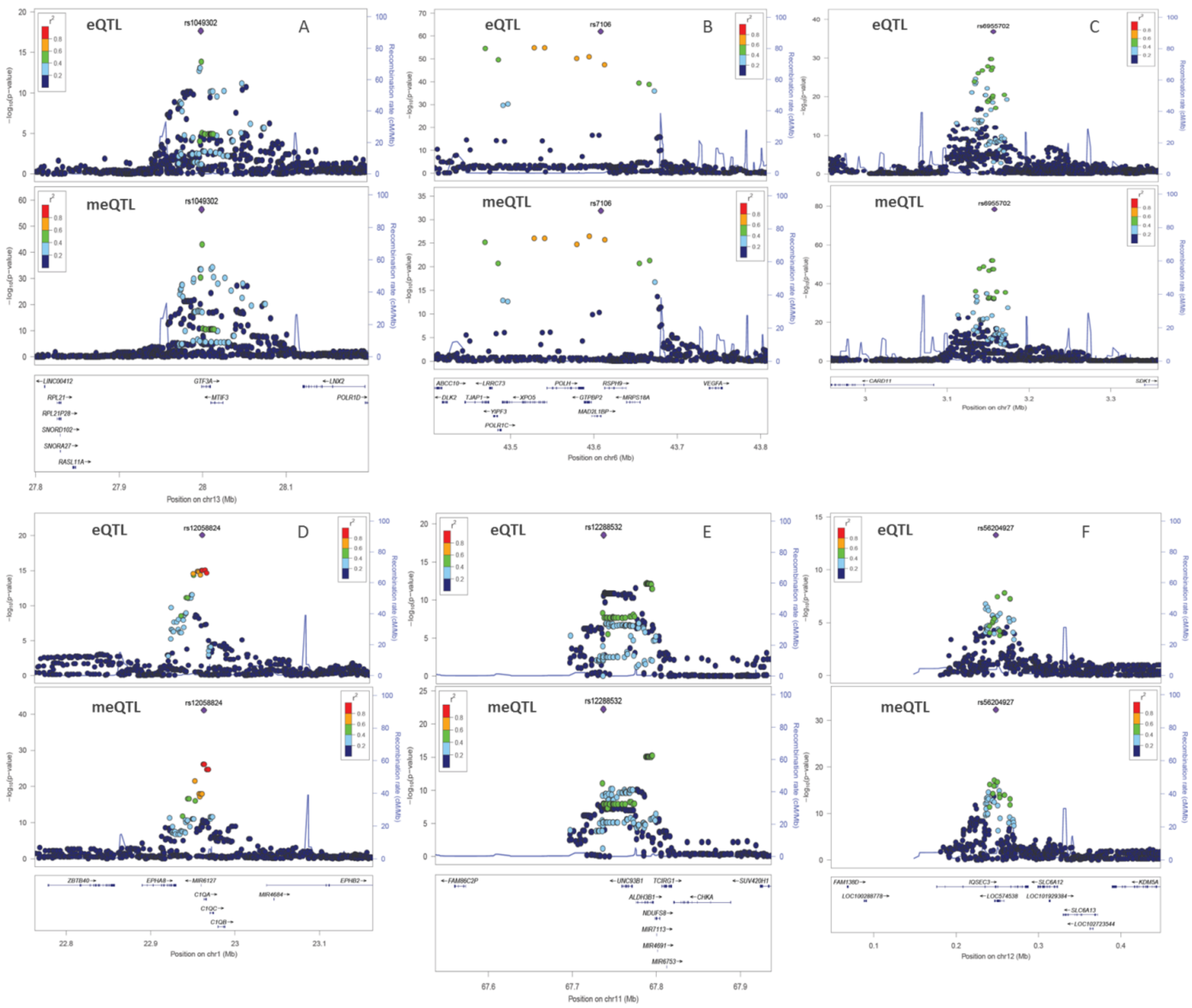

Supplementary Figure 4. Locus zoom plots for 6 examples of co-localized eQTL-meQTL pairs in Figure 3. The color of the dots in the eQTL plots reflects the LD r^2 level to the lead meSNP for the corresponding CpG site and the color of the dots in the meQTL plots reflects the LD r^2 level to the lead eSNP for the corresponding gene.

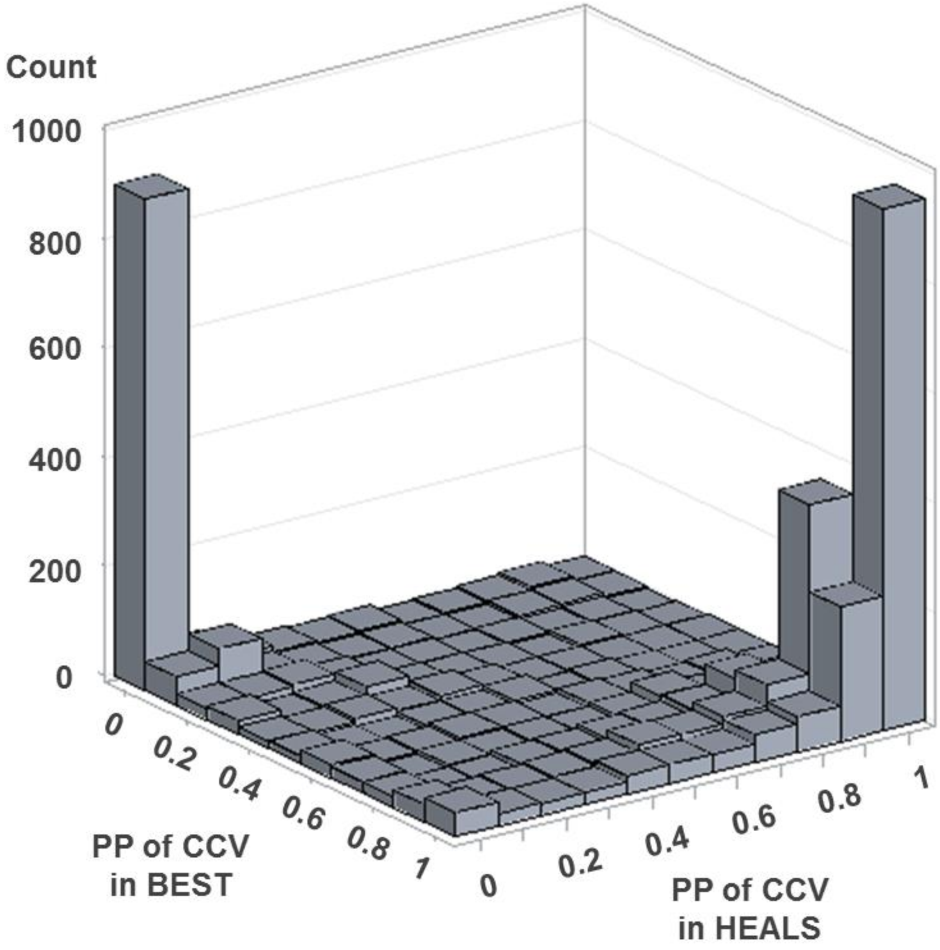

Supplementary Figure 5. Results of replication of co-localization analyses for 4,875 eQTL-meQTL pairs. The histogram compares the posterior probability (PP) of a common causal variant (CCV) based on BEST methylation data (450K array) to PP of CCV based on HEALS methylation data (EPIC array). Both analyses use a p_{12} value of $4.4x10^{-4}$.

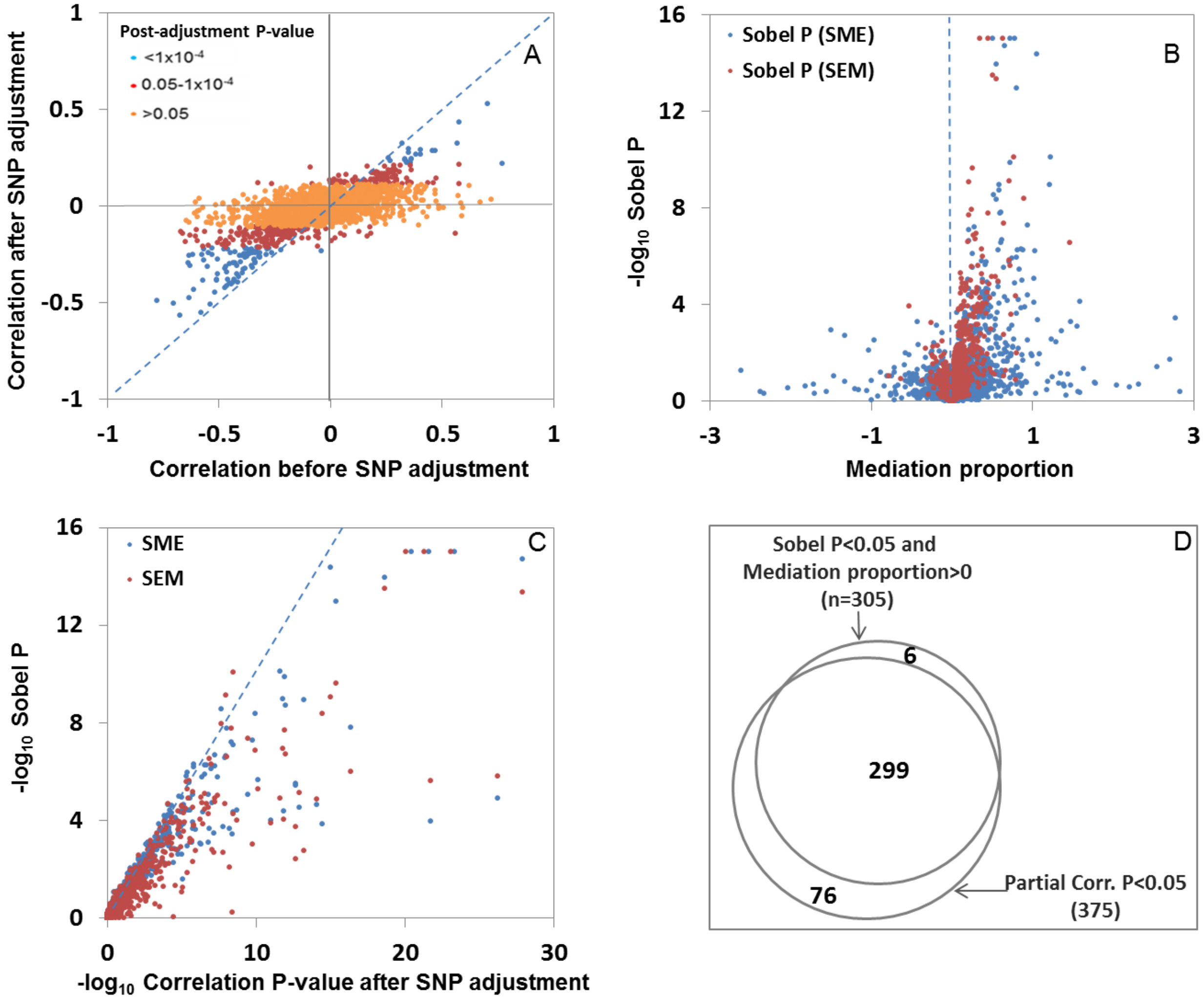

Supplementary Figure 6. Partial correlation and mediation analyses provide evidence for shared a regulatory mechanism (p_{12}=2.9x10^{-4}). Model for each of 2,098 potentially co-localized eQTL-mQTL pairs (using p_{12}=2.9x10^{-4}) includes adjustments for age, sex, and PCs from both the expression and methylation data (n=316). A: results from partial correlation analysis. B: Mediation analysis results for the SME and the SEM model. Mediation proportion outliers out of -3 to 3 ranges were removed from the figure and Sobel P outliers <10^{-15} were set to the 10^{-15}. C: Relationship between Sobel P from mediation analyses and the post-adjustment correlation P values from partial correlation analysis. Sobel P outliers <10^{-15} were set to the 10^{-15}. D: Venn diagram showing the concordance between mediation analysis and partial correlation analyses.

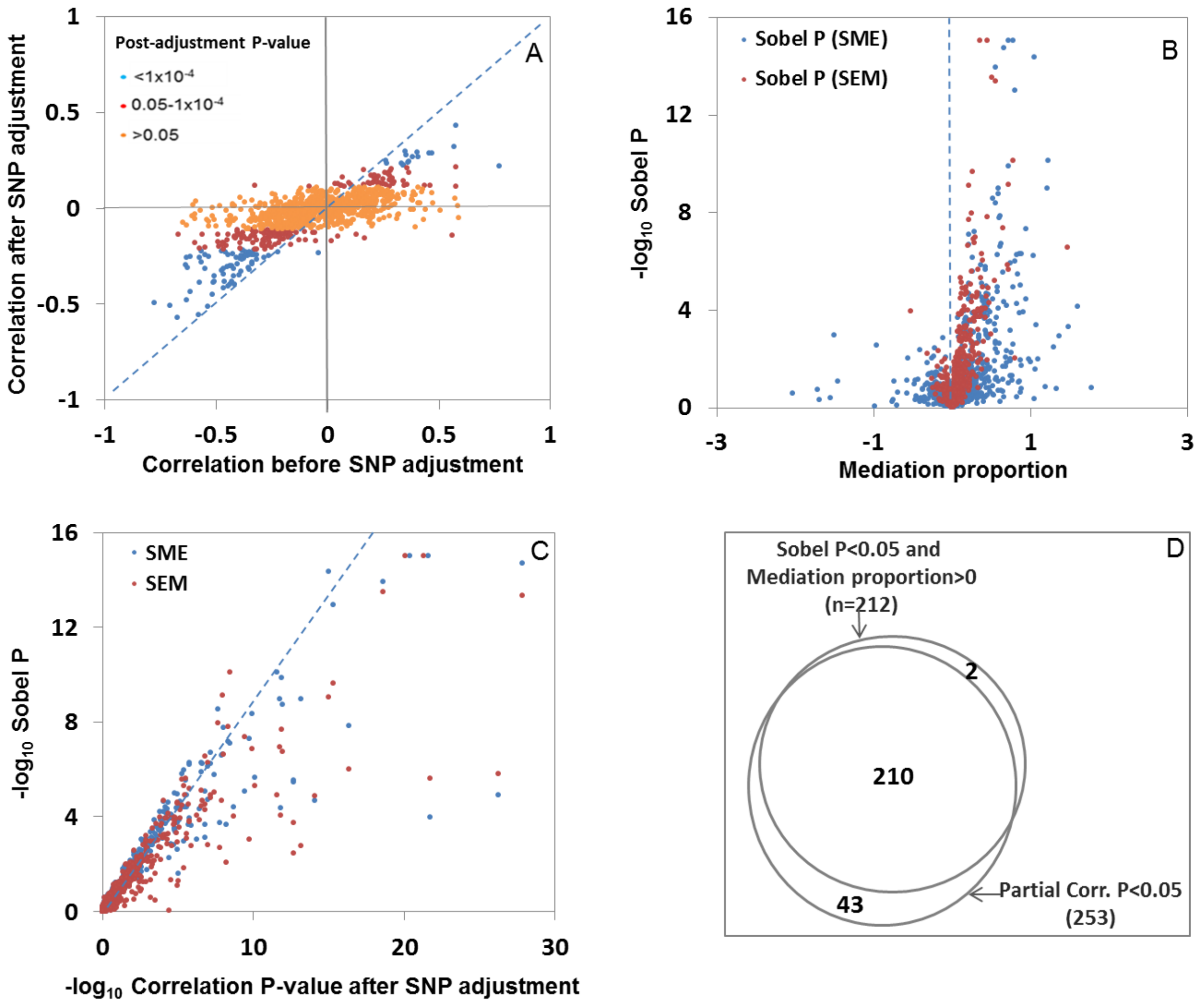

Supplementary Figure 7. Partial correlation and mediation analyses provide evidence for shared a regulatory mechanism (p_{12}=1.45x10^{-4}). Model for each of 1,047 potentially co-localized eQTL-mQTL pairs (using p_{12}=1.45x10^{-4}) includes adjustments for age, sex, and PCs from both the expression and methylation data (n=316). A: results from partial correlation analysis. B: Mediation analysis results for the SME and the SEM model. Mediation proportion outliers out of -3 to 3 ranges were removed from the figure and Sobel P outliers <10^{-15} were set to the 10^{-15}. C: Relationship between Sobel P from mediation analyses and the post-adjustment correlation P values from partial correlation analysis. Sobel P outliers <10^{-15} were set to the 10^{-15}. D: Venn diagram showing the concordance between mediation analysis and partial correlation analyses.

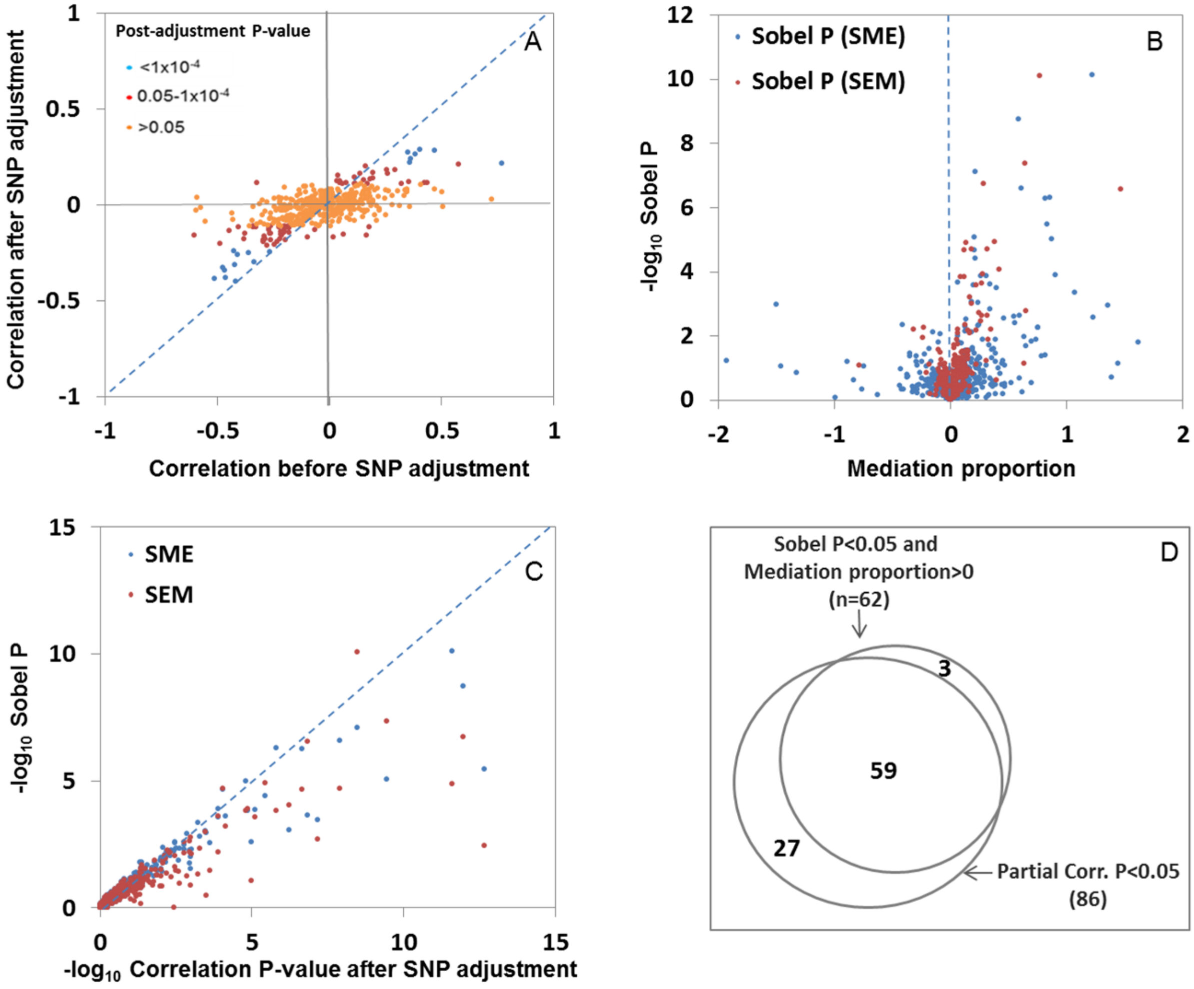

Supplementary Figure 8. Partial correlation and mediation analyses provide evidence for shared a regulatory mechanism (p_{12}=5.8x10^{-5}). Model for each of 473 potentially co-localized eQTL-mQTL pairs includes adjustments for age, sex, and PCs from both the expression and methylation data (n=316). A: results from partial correlation analysis. B: Mediation analysis results for the SME and the SEM model. C: Relationship between Sobel P from mediation analyses and the post-adjustment correlation P values from partial correlation analysis. D: Venn diagram showing the concordance between mediation analysis and partial correlation analyses.

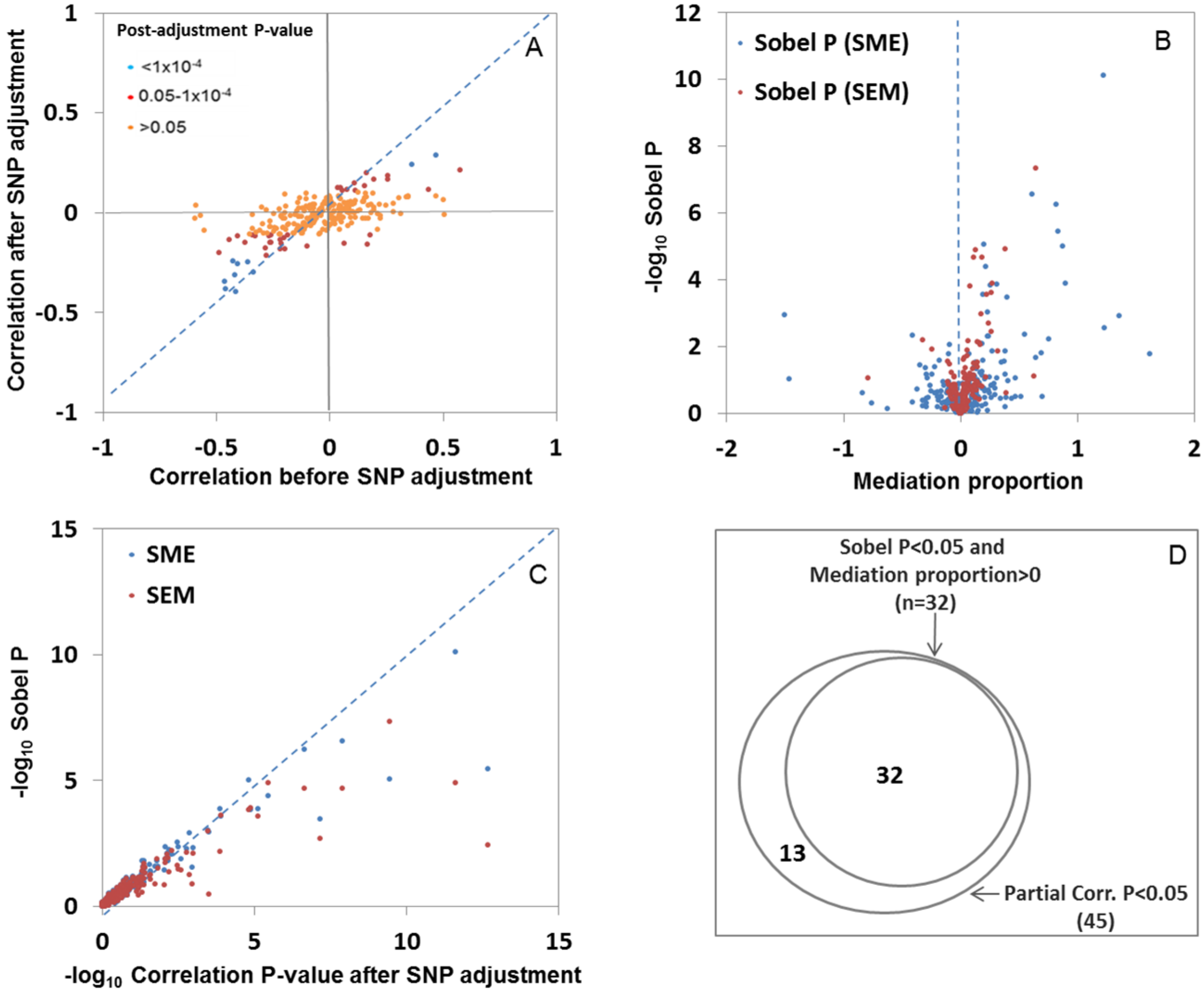

Supplementary Figure 9. Partial correlation and mediation analyses provide evidence for shared a regulatory mechanism (p_{12}=2.9x10^{-5}). Model for each of 266 potentially co-localized eQTL-mQTL pairs includes adjustments for age, sex, and PCs from both the expression and methylation data (n=316). A: results from partial correlation analysis. B: Mediation analysis results for the SME and the SEM model. C: Relationship between Sobel P from mediation analyses and the post-adjustment correlation P values from partial correlation analysis. D: Venn diagram showing the concordance between mediation analysis and partial correlation analyses.

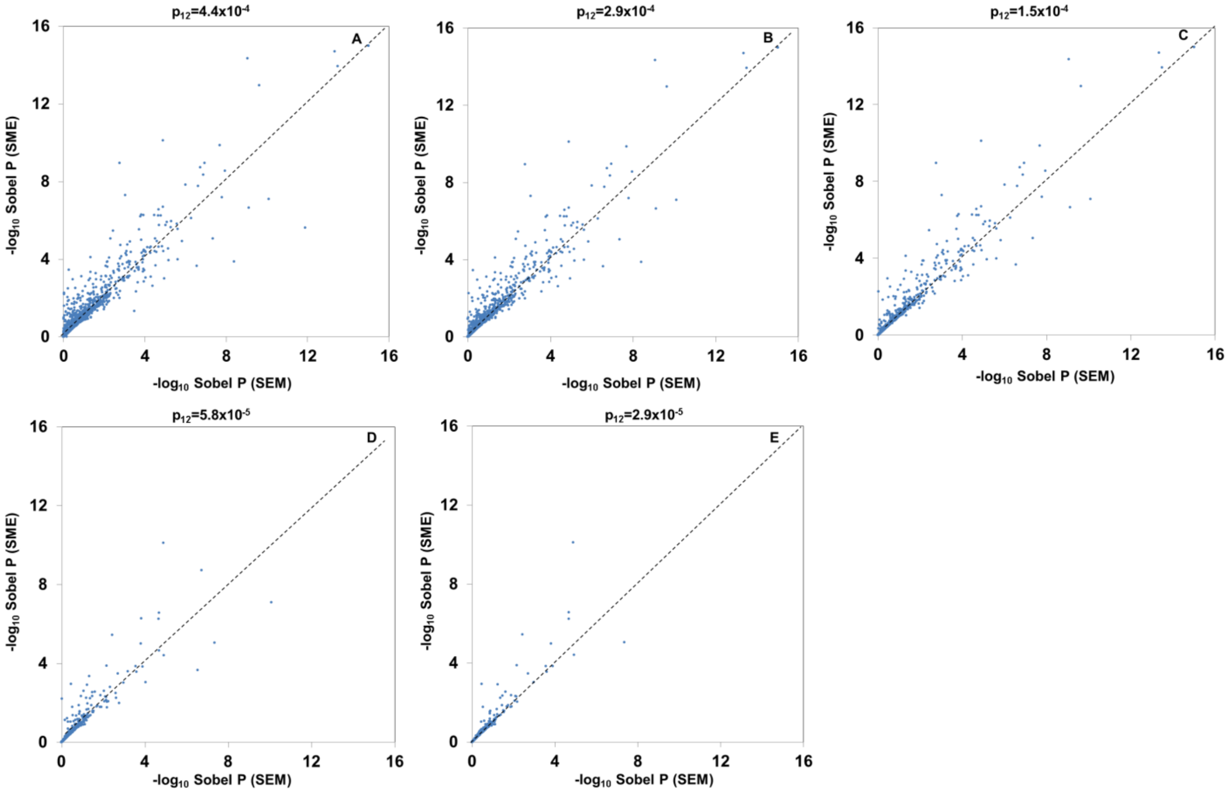

Supplementary Figure 10. Scatter plots of Sobel P values for SME and SEM models. A: p_{12}=4.4x10^{-4}; B: p_{12}=2.9x10^{-4}, C: p_{12}=1.45x10^{-4}, D: p_{12}=2.8x10^{-5}, E: p_{12}=2.9x10^{-5}

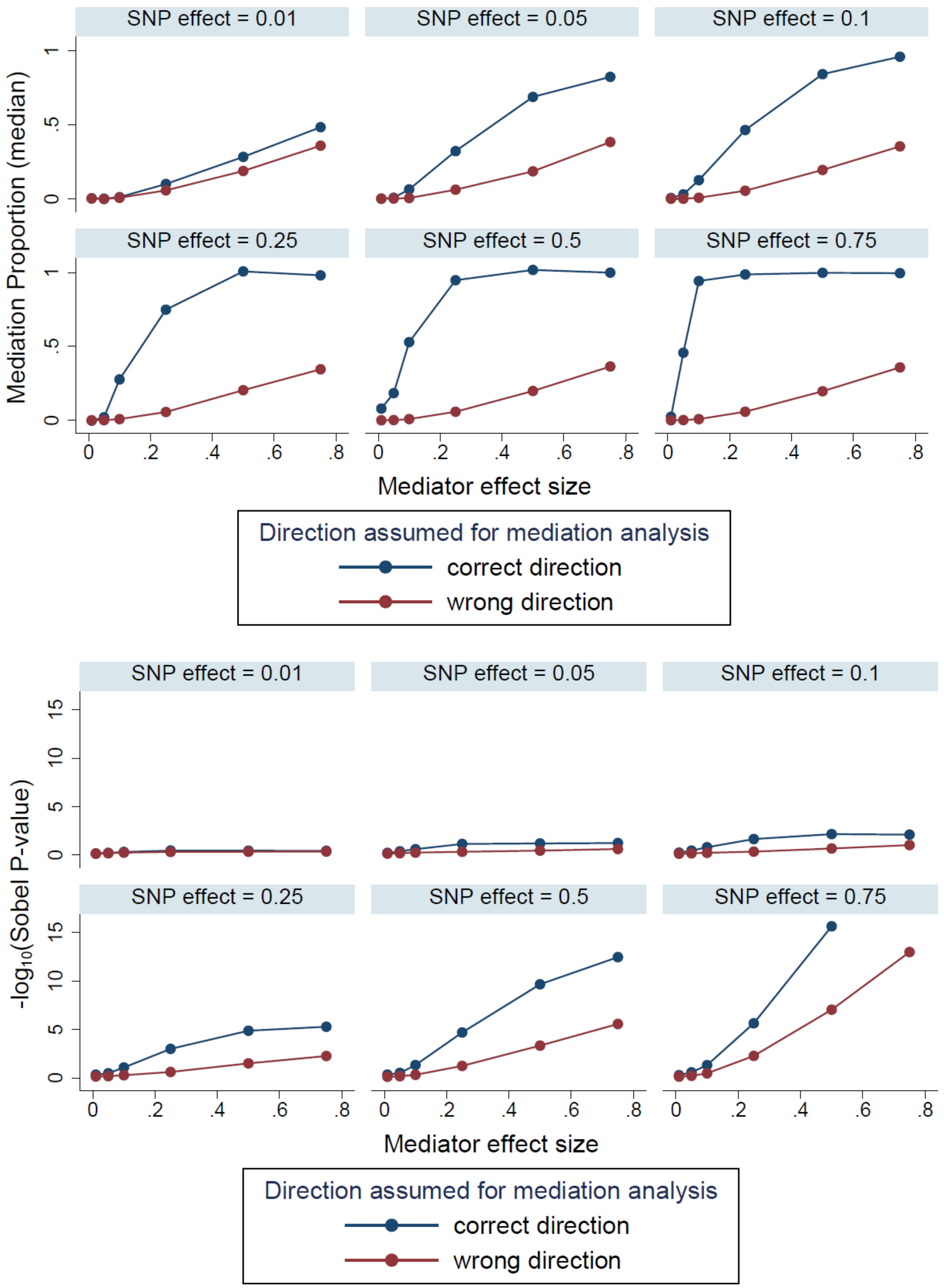

Supplementary Figure 11. Evidence for mediation will be stronger when the correct model is specified. Results are based on simulated data of sample size 316. The top panel reports the mediation proportion and the bottom panel reports the $-\log_{10}$(Sobel P), varying the effect sizes of the SNP and the mediator.

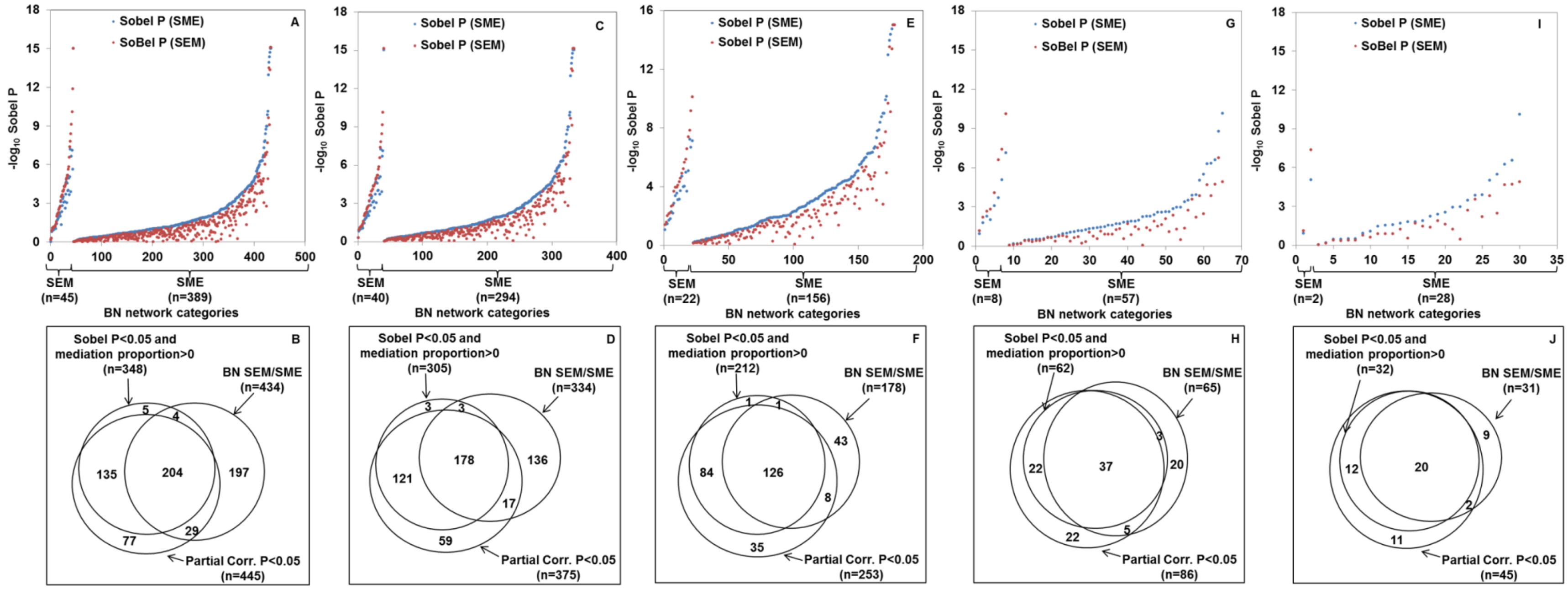

Supplementary Figure 12. Results from mediation analysis compared with Bayesian Network and partial correlation analyses. Upper panel: Sobel P values plotted according to the causal model selected by Bayesian network analyses. Lower panel: Venn Diagram demonstrating concordance among three methods for assessing evidence for a shared regulatory mechanism. Plot A and B: $p_{12}=4.4x10^{-4}$,Plot C and D: $p_{12}=2.9x10^{-4}$, Plot E and F: $p_{12}=1.5x10^{-4}$, Plot G and H: $p_{12}=5.8x10^{-5}$, Plot I and J: $p_{12}=2.9x10^{-5}$.

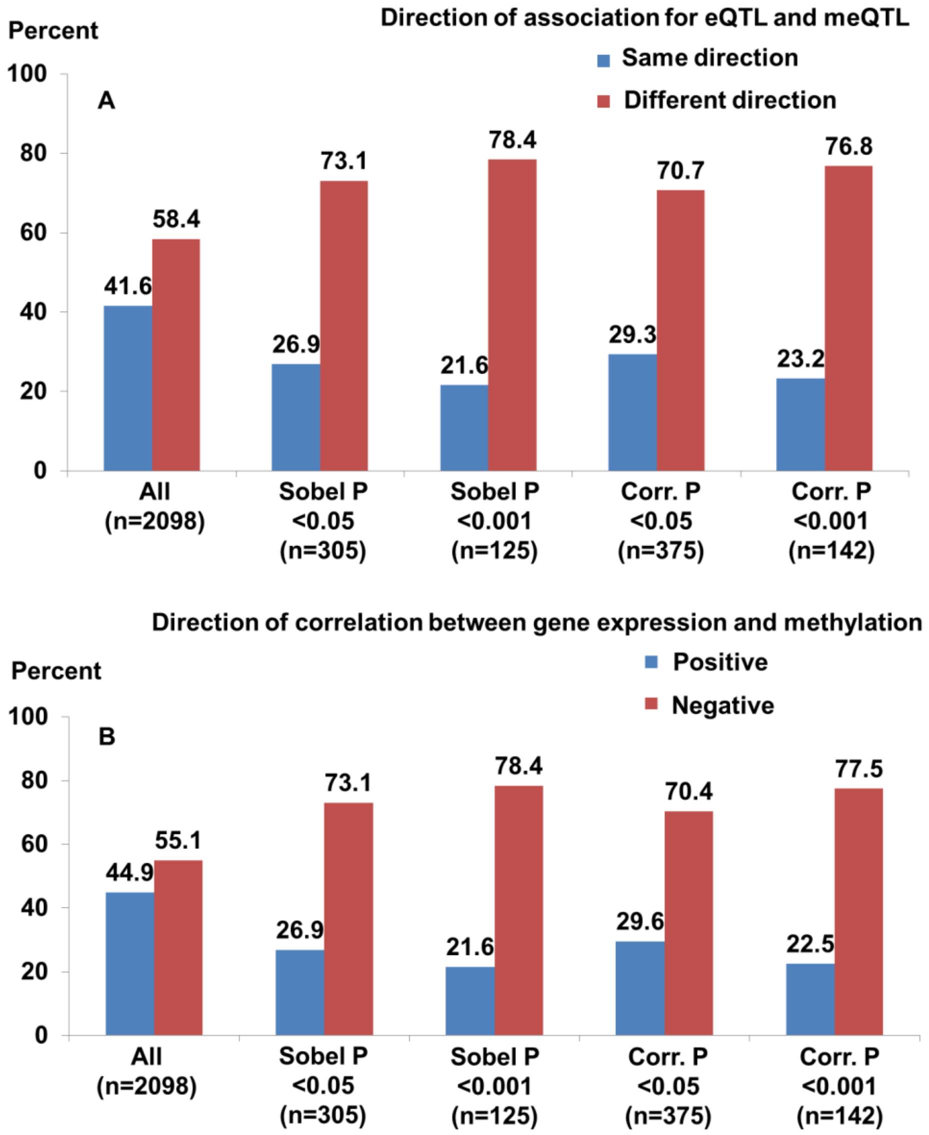

Supplementary Figure 13. Direction of QTL effects and associations between expression and methylation for co-localized eQTL-meQTL pairs. Co-localized pairs using a p_{12} value $2.9\text{x}10^{-4}$ are presented. Results are stratified according to P-values from mediation analysis (Sobel P) and partial correlation analysis (Corr. P). A: Histograms of the percentage of eQTL-meQTL pairs showing the same or different direction of association. B: Histograms of the percentage of eQTL-meQTL pairs for which the direction of association between gene expression and DNA methylation is positive or negative.

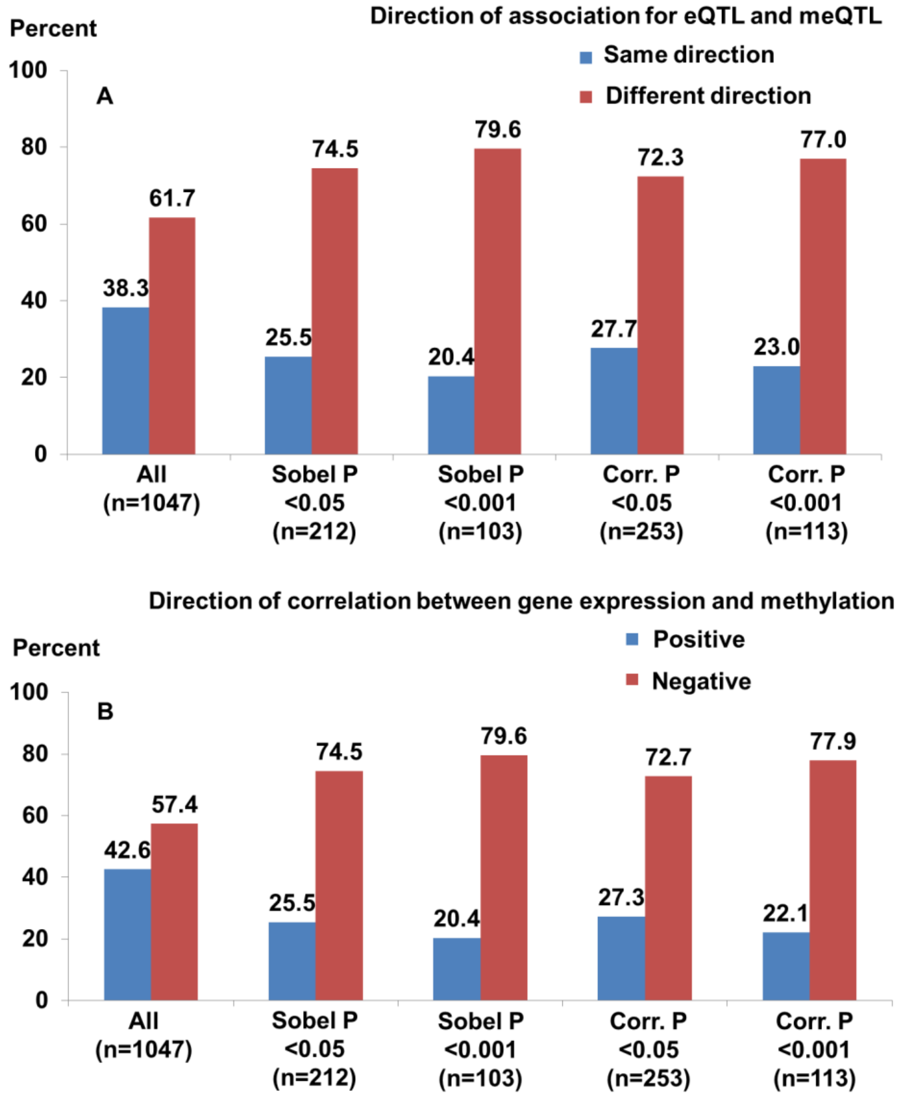

Supplementary Figure 14. Direction of QTL effects and associations between expression and methylation for co-localized eQTL-meQTL pairs. Co-localized pairs using a p_{12} value $1.45x10^{-4}$ are presented. Results are stratified according to P-values from mediation analysis (Sobel P) and partial correlation analysis (Corr. P). A: Histograms of the percentage of eQTL-meQTL pairs showing the same or different direction of association. B: Histograms of the percentage of eQTL-meQTL pairs for which the direction of association between gene expression and DNA methylation is positive or negative.

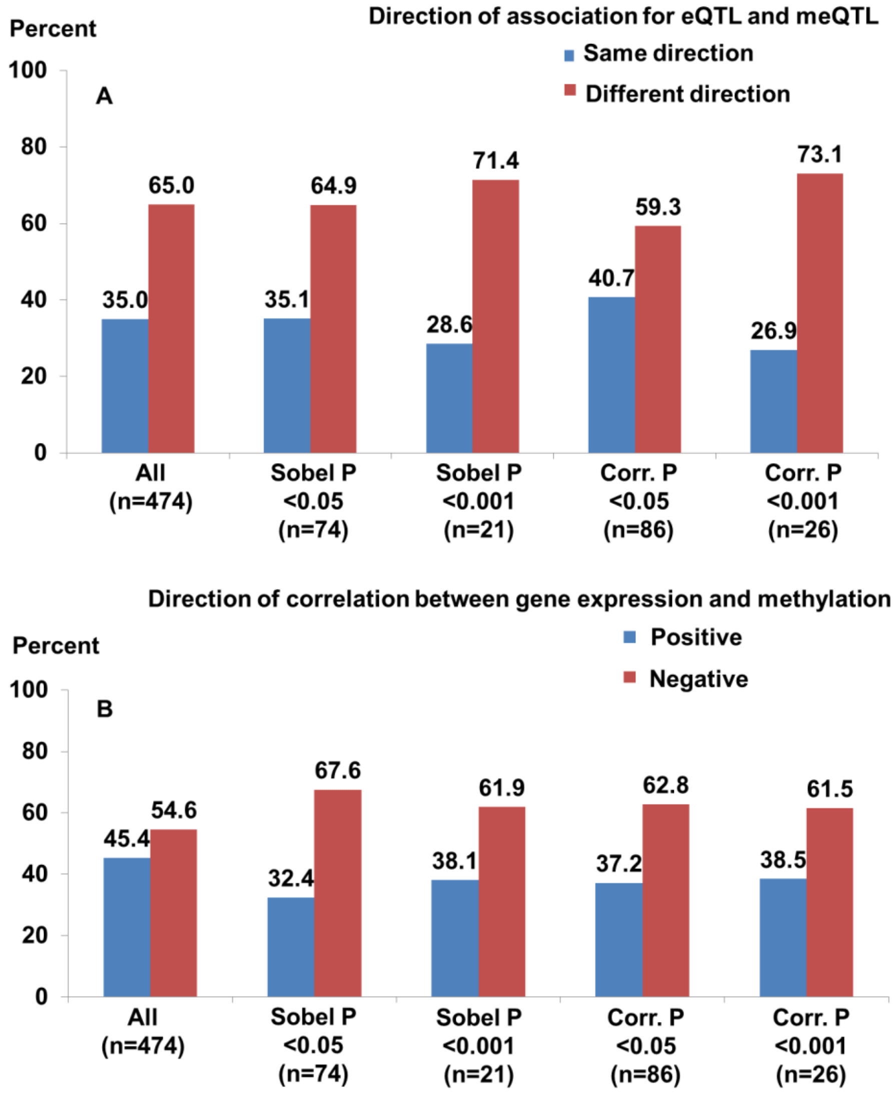

Supplementary Figure 15. Direction of QTL effects and associations between expression and methylation for co-localized eQTL-meQTL pairs. Co-localized pairs using a p_{12} value $5.8x10^{-5}$ are presented. Results are stratified according to P-values from mediation analysis (Sobel P) and partial correlation analysis (Corr. P). A: Histograms of the percentage of eQTL-meQTL pairs showing the same or different direction of association. B: Histograms of the percentage of eQTL-meQTL pairs for which the direction of association between gene expression and DNA methylation is positive or negative.

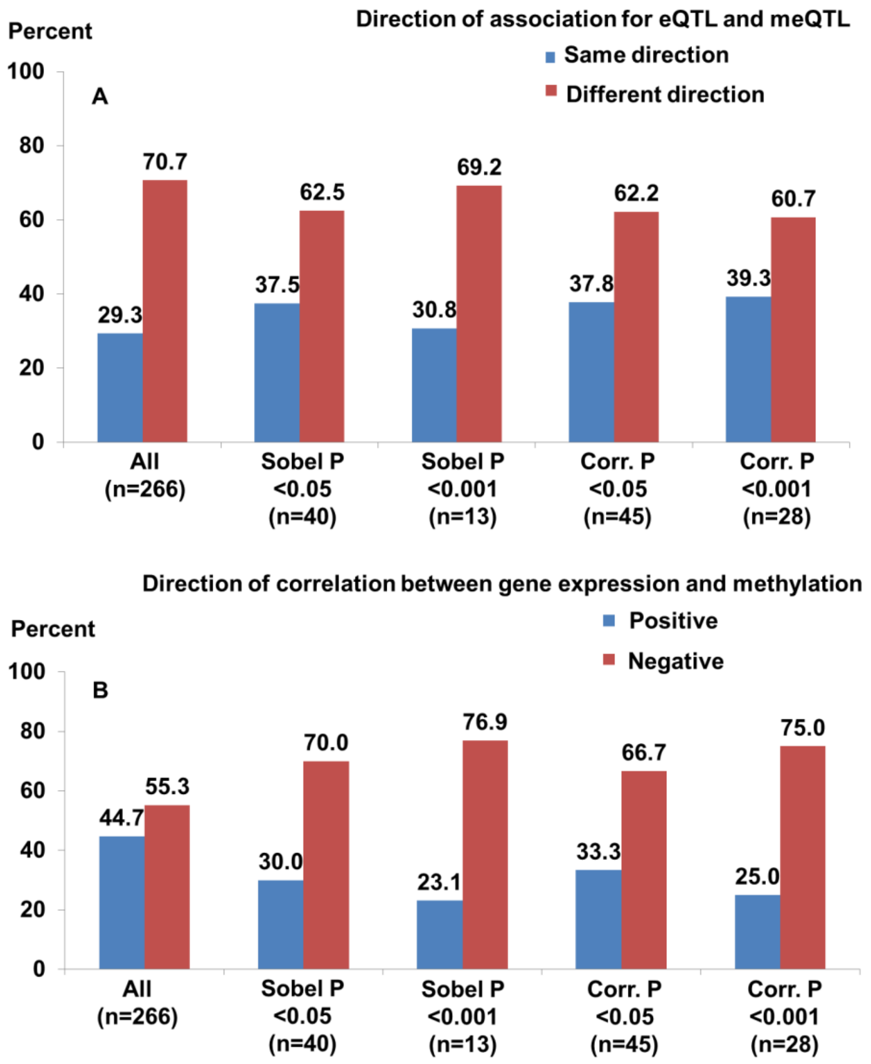

Supplementary Figure 16. Direction of QTL effects and associations between expression and methylation for co-localized eQTL-meQTL pairs. Co-localized pairs using a p_{12} value $2.9x10^{-5}$ are presented. Results are stratified according to P-values from mediation analysis (Sobel P) and partial correlation analysis (Corr. P). A: Histograms of the percentage of eQTL-meQTL pairs showing the same or different direction of association. B: Histograms of the percentage of eQTL-meQTL pairs for which the direction of association between gene expression and DNA methylation is positive or negative.

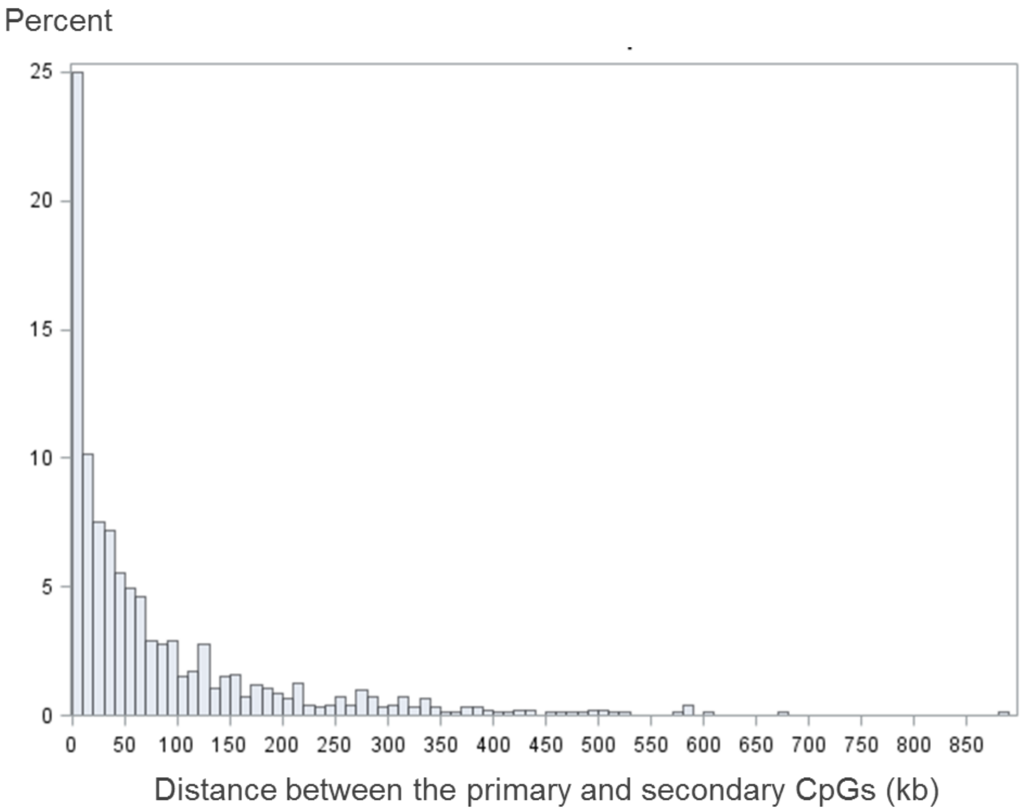

Supplementary Figure 17. Distribution of distance between the primary and secondary CpGs for n=955 cases with expression and primary methylation in the same direction but have at least one secondary CpG inversely associated with the CpG originally selected.

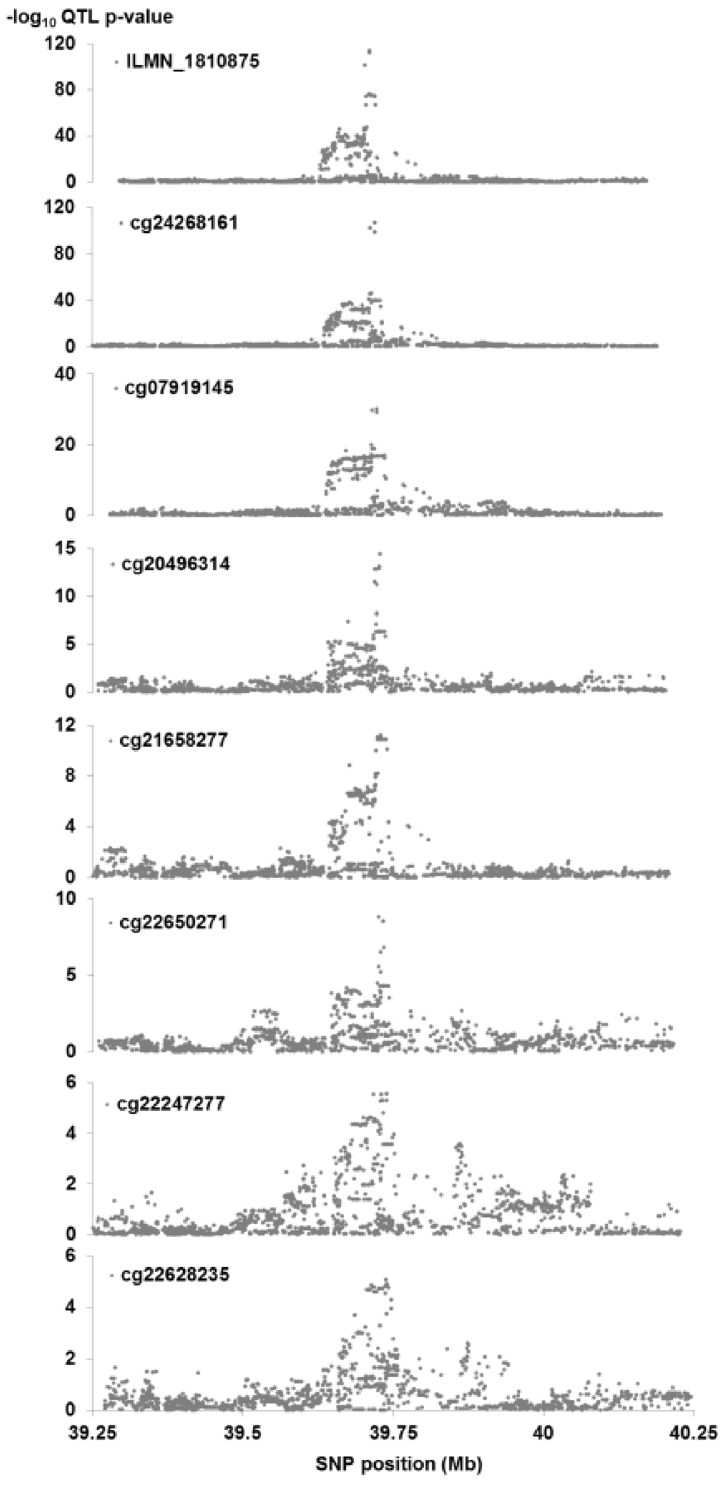

Supplementary Figure 18. Regional association plots for one eQTL affecting *SYNGR1* (ILMN_1810875) a co-localizing meQTL that affects seven nearby CpGs. The genomic region to which these association plots correspond is shown in Figure 8.

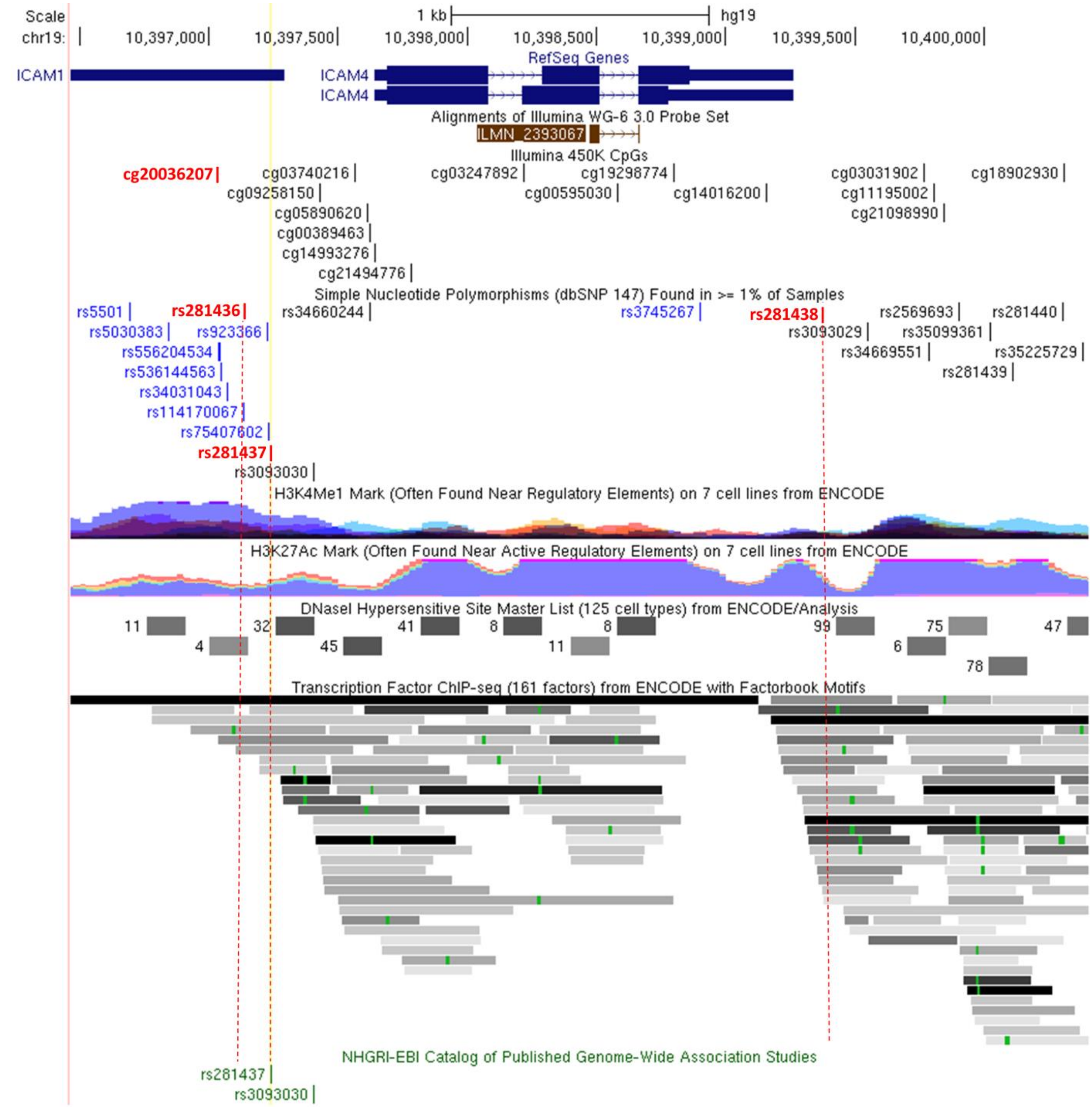

Supplementary Figure 19. Overlap with genomic annotations for three candidate causal SNPs for a co-localized eQTL-meQTL pair that shows strong evidence of mediation. Expression of the ICAM4 gene and methylation at nearby CpG cg20036207 (in red) are affected by a common causal variant. SNP rs281437 was the lead *cis*-eSNP for ILMN_2393067 ($P=10^{-23}$) which captures expression of all RefSeq isoforms of ICAM4. This eQTL co-localizes with a meQTL affecting methylation at cg20036207 (Probability of CCV>99%), with strong evidence of partial correlation (residual r= -0.30, $P=5x10^{-8}$) and mediation (SEM P = $2x10^{-5}$; SME P= $6x10^{-7}$). This SNP is in strong LD (r^2>0.65) with only two SNPs in the Bangladeshi population (1KG BEB), rs281436 (r^2=0.97) and rs281438 (r^2=0.77). The two SNPs showing the strongest association with methylation AND expression, rs281437 and rs281436, reside 107 bp apart, and rs281436 is ~ 100 bp from cg20036207.

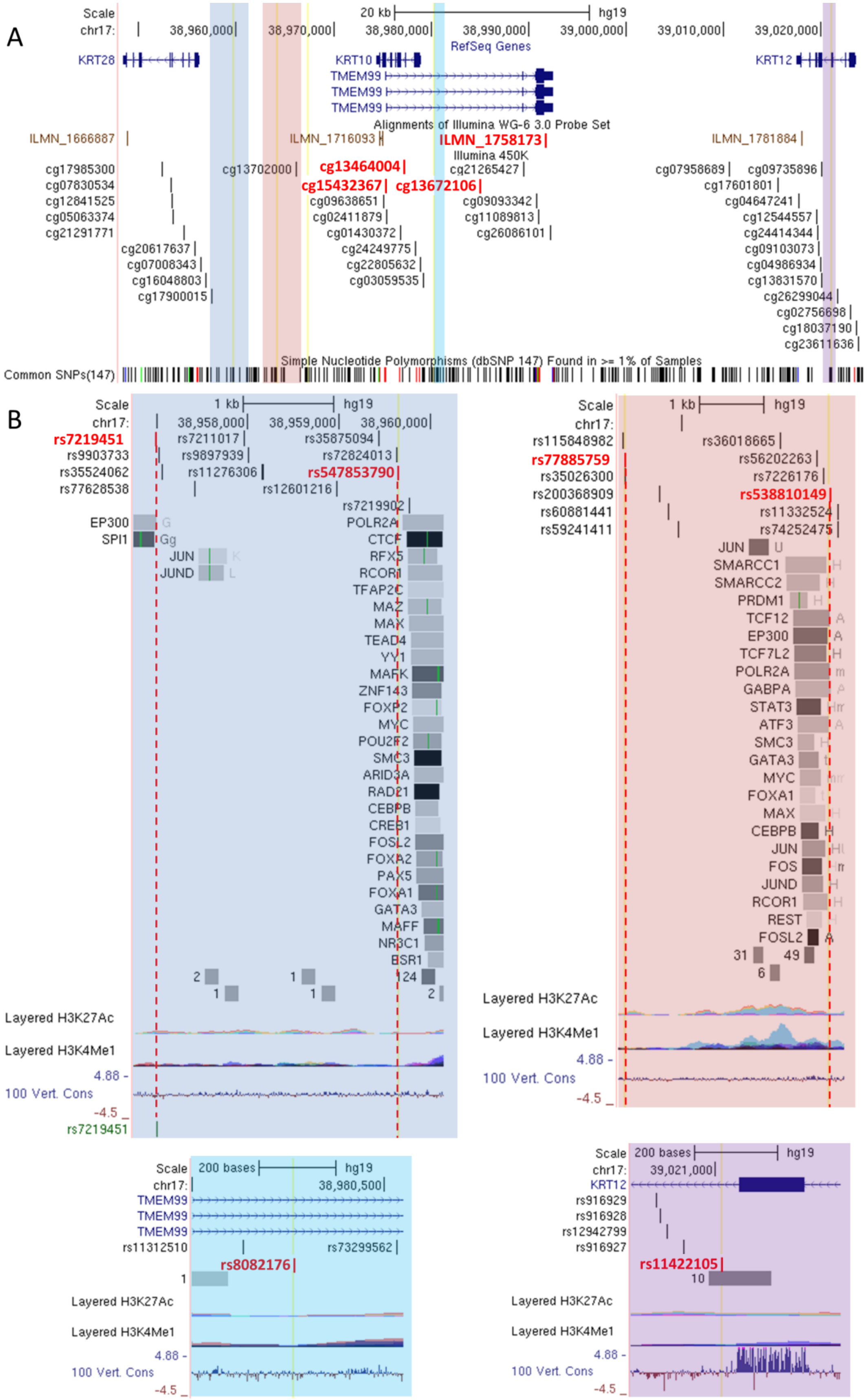
A
Scale
20 kb
hg19
chr17:
38,960,000
38,970,000
38,980,000
38,990,000
39,000,000
39,010,000
39,020,000
RefSeq Genes
KRT28
KRT10
TMEM99
TMEM99
TMEM99
KRT12
Alignments of Illumina WG-6 3.0 Probe Set
ILMN_1666887
ILMN_1716093
ILMN_1758173
ILMN_1781884
Illumina 450K
cg17985300
cg07830534
cg12841525
cg05063374
cg21291771
cg20617637
cg07008343
cg16048803
cg17900015
cg13702000
cg13464004
cg15432367
cg13672106
cg09636651
cg02411879
cg01430372
cg24249775
cg22805632
cg03059535
cg21265427
cg09093342
cg11089813
cg26086101
cg07958689
cg09735896
cg17601801
cg04647241
cg12544557
cg24414344
cg09103073
cg04986934
cg13831570
cg26299044
cg02756698
cg18037190
cg23611636
Simple Nucleotide Polymorphisms (dbSNP 147) Found in >= 1% of Samples
Common SNPs(147)
B
Scale
1 kb
hg19
chr17:
38,958,000
38,959,000
38,960,000
rs7219451
rs9903733
rs35524062
rs77628538
rs7211017
rs9897939
rs11276306
rs12601216
rs35875094
rs72824013
rs547853790
rs7219902
EP300
SPI1
JUN
JUND
POLR2A
CTCF
RFX5
RCOR1
TFAP2C
MAZ
MAX
TEAD4
YY1
MAFK
ZNF143
FOXP2
MYC
POU2F2
SMC3
ARID3A
RAD21
CEBPB
CREB1
FOSL2
FOXA2
PAX5
FOXA1
GATA3
MAFF
NR3C1
ESR1
Layered H3K27Ac
Layered H3K4Me1
4.88
100 Vert. Cons
-4.5
rs7219451
Scale
1 kb
hg19
chr17:
rs115848982
rs77885759
rs35026300
rs200368909
rs60881441
rs59241411
rs36018665
rs56202263
rs7226176
rs538810149
rs11332524
rs74252475
JUN
SMARCC1
SMARCC2
PRDM1
TCF12
EP300
TCF7L2
POLR2A
GABPA
STAT3
ATF3
SMC3
GATA3
MYC
FOXA1
MAX
CEBPB
JUN
FOS
JUND
RCOR1
REST
FOSL2
Layered H3K27Ac
Layered H3K4Me1
4.88
100 Vert. Cons
-4.5
Scale
200 bases
hg19
chr17:
38,980,500
TMEM99
TMEM99
TMEM99
rs11312510
rs73299562
rs8082176
Layered H3K27Ac
Layered H3K4Me1
4.88
100 Vert. Cons
-4.5
Scale
200 bases
hg19
chr17:
39,021,000
KRT12
rs916929
rs916928
rs12942799
rs916927
rs11422105
Layered H3K27Ac
Layered H3K4Me1
4.88
100 Vert. Cons
-4.5

Supplementary Figure 20. Overlap with genomic annotations for six candidate causal SNPs for a co-localized eQTL-meQTL pair that shows strong evidence of mediation. Insertion rs538810149 was the lead *cis*-eSNP for expression probe ILMN_1758173 ($P=10^{-24}$) which captures expression of KMEM99 (Panel A). This eQTL co-localized with a meQTL associated with methylations at 3 CpG sites: cg13464004, cg15432367, and cg13672106 (Panel A). We observed strong evidence of partial correlation (residual r = -0.35; $P=3x10^{-10}$) and mediation (SEM $P=7x10^{-4}$; SME $P=5x10^{-8}$). This SNP is in strong LD with rs547853790 ($r^2=0.93$), rs7219451 ($r^2=0.93$), rs35026300 ($r^2=0.93$), rs8082176 ($r^2=0.92$), and rs11422105 ($r^2=0.86$). Panel B shows overlap of candidate causal variants with transcription factor binding sites, DNAse-I hypersensitivity sites, histone marks, and conversation among vertebrates, and GWAS SNPs (in that order). Panel B regions are color-coded to match the regions highlighted in Panel A. None of these SNPs show striking overlap with any of the regulatory features examined, other than rs11422105 which resides within a 10-tissue DNase-I hypersensitivity site.
