# Supplementary material for: Co-occurring expression and methylation QTLs allow detection of common causal variants and shared biological mechanisms
Source: Nat Commun. 2018 Feb 23;9:804. doi: 10.1038/s41467-018-03209-9 (PMC5824840; doi:10.1038/s41467-018-03209-9)
Supplement: Supplementary file 3 — Description of Additional Supplementary Files [file 41467_2018_3209_MOESM3_ESM.pdf]

**Description of Additional Supplementary Files**

**File Name: Supplementary Data 1**
**Description:** Characteristics of eQTL-meQTL pairs and results of co-localization analyses.

**File Name: Supplementary Data 2**
**Description:** Linear regression of the posterior probabilities of common causal variant on lead eSNP LD-score and the eQTL/meQTL P-values.

**File Name: Supplementary Data 3**
**Description:** Results from mediation analyses and partial correlation analyses.

**File Name: Supplementary Data 4**
**Description**: Results from partial correlation analyses adjusting for top two lead SNPs.
